# Pre-operative embolization facilitating a posterior approach for the surgical resection of giant sacral neurogenic tumors

**DOI:** 10.3892/ol.2013.1328

**Published:** 2013-05-08

**Authors:** KANGWU CHEN, MING ZHOU, HUILIN YANG, ZHONGLAI QIAN, GENLIN WANG, GUIZHONG WU, XIAOYU ZHU, ZHIYONG SUN

**Affiliations:** Department of Orthopedic Surgery, The First Affiliated Hospital of Soochow University, Suzhou, Jiangsu 215006, P.R. China

**Keywords:** embolization, neurogenic, sacrum, schwannoma, surgery

## Abstract

The present study aimed to assess a posterior approach for the surgical resection of giant sacral neurogenic tumors, and to evaluate the oncological and functional outcomes. A total of 16 patients with giant sacral neurogenic tumors underwent pre-operative embolization and subsequent posterior sacral resection between January 2000 and June 2010. Benign tumors were identified in 12 cases, while four cases exhibited malignant peripheral nerve sheath tumors (MPNSTs). An evaluation of the operative techniques used, the level of blood loss, any complications and the functional and oncological outcomes was performed. All tumor masses were removed completely without intra-operative shock or fatalities. The mean tumor size was 17.5 cm (range, 11.5–28 cm) at the greatest diameter. The average level of intra-operative blood loss was 1,293 ml (range, 400–4,500 ml). Wound complications occurred in four patients (25%), including three cases of cutaneous necrosis and one wound infection. The mean follow-up time was 59 months (range, 24–110 months). Tumor recurrence or patient mortality as a result of the disease did not occur in any of the patients with benign sacral neurogenic tumors. The survival rate of the patients with malignant lesions was 75% (3/4 patients) since 25 % (1/4 patients) had multiple local recurrences and succumbed to the disease. The patients with benign tumors scored an average of 92.8% on the Musculoskeletal Tumor Society (MSTS) score functional evaluation, while the patients with malignant tumors scored an average of 60.3%. A posterior approach for the surgical resection of giant sacral neurogenic tumors, combined with pre-operative embolization may be safely conducted with satisfactory oncological and functional outcomes.

## Introduction

Giant sacral neurogenic tumors are rare lesions which include schwannomas, neurofibromas and malignant peripheral nerve sheath tumors (MPNSTs), and are estimated to represent ∼10% of presacral tumors (1). The tumors usually grow towards the presacral space and present with extremely large dimensions (2). Previous studies on the tumors are limited and consist of a relatively small number of cases (2–5). Giant sacral neurogenic tumors are characterized by an indolent growth pattern and non-specific symptoms (6). Although the majority of these tumors are benign, they may become locally aggressive, cause catastrophic neurological impairment and weaken pelvic arch stability. The initial treatment of a tumor by complete resection may give a patient the best chance of an overall and disease-free survival (7). However, surgical resection is complex due to the anatomical characteristics of the region, the extent of the tumor, the substantial blood loss during surgery and the propensity for local recurrence (6,7). The requirement for adequate tumor removal must be balanced against the preservation of nerve function. Yang *et al* (8) identified that pre-operative arterial embolization resulted in decreased intra-operative blood loss and allowed a more aggressive approach to the complete resection of the tumors. In the present study, the use of a posterior approach following transcatheter arterial embolization was assessed in the complete resection of giant sacral neurogenic tumors. Furthermore, the oncological outcomes and Musculoskeletal Tumor Society (MSTS) scores were evaluated.

## Patients and methods

### Patients

The records of 16 patients with giant sacral neurogenic tumors who underwent surgical excision in The First Affiliated Hospital of Soochow University (Suzhou, Jiangsu, China) between January 2000 and June 2010 were identified and retrospectively analyzed. The patients included 7 males and 9 females, with a mean age of 39.9 years (range, 17–62 years). Previous surgical treatments had been provided to three patients at other hospitals. The duration between the onset and presentation of the symptoms was 1–252 months (average, 94 months). Pain in the sacrococcygeal region was the most common presenting symptom, followed by rectal dysfunction or urinary disturbance (Table I). The tumors were detected as firm presacral masses by a clinical rectal examination in nine of the patients. All patients received a histological diagnosis using a computerized tomography-guided percutaneous needle biopsy. A total of 12 patients (75%) had benign tumors, including 10 schwannomas and 2 neurofibromas. MPNSTs were identified in four patients (25%). Plain X-ray, computed tomography (CT) and magnetic resonance imaging (MRI) scans were obtained pre-operatively. In the high sacrum (above S3), 11 tumors were located. The remaining five were situated in the low sacrum (S3 or below). The tumor size was recorded as the maximum tumor dimension measured on the gross specimen or on the pre-operative MRI. Approval for the present study was obtained from the ethics committee of Soochow University and all patients provided their informed consent.

The management of the giant sacral neurogenic tumors was performed by a single approach involving a multidisciplinary team that consisted of two or more surgical specialists, including orthopedic surgeons, neurosurgeons, colorectal surgeons, urologists, gynecologists and plastic surgeons. The team created an appropriate surgical plan with the goal of obtaining adequate margins.

### Surgical procedure

All patients underwent pre-operative embolization of the tumor-feeding arteries under digital subtraction angiography (DSA; Siemens Angiostar Plus, Seimens, Munich, Germany). The bilateral internal iliac, median sacral and other small tumor-feeding arteries were embolized using gelatin sponges, ranging in size from a strip to a particle. The angiogram was then performed again across the abdominal aorta to ensure that all tumor-supplying vessels were embolized.

The surgical excision of the tumor was performed within 48 h following the embolization. A pre-operative bowel preparation and urethral catheterization were performed in all patients. With the patient in the prone position, and once the rectum had been identified by palpating the anal tube, the posterior ‘I’ incision was performed. The approach offered a good exposure of the sacrum, the dorsal sections of the iliac wings and the surrounding soft tissues, while allowing exposure of the upper lumbar spine where necessary. The posterior, caudal and bilateral aspects of the tumor were excised using wide or marginal approaches, while the anterior aspect was separated by blunt dissection through the retroperitoneal interstitial space, with care being taken to ensure that the integrity of the wall was not violated. Palpating the anal tube was an efficient way to certify the location of the rectum. The anterior aspect of the sacrum was exposed by resecting parts of the iliac bone where necessary. The bilateral S1–S2 nerve roots, or at least one S3 nerve root, were preserved in the patients with benign tumors. In the instances where a nerve root penetrated the tumor, the membrane of this nerve was dissected. For the patients with malignant neurogenic tumors, the affected sacral nerves were sacrificed in order to achieve adequate margins. The dura was ligated twice using a silk suture to avoid a dural leak. Where the S1 segment, total sacrum and substantial parts of the iliac wings were excised, the stability of the pelvic ring was reconstructed using pedicle screws, iliac screws and lumbopelvic rods.

The minimum duration of patient follow-up was two years subsequent to the surgery. All data was obtained from available records or a telephone interview. The follow-up procedure included plain radiograph, CT and MRI evaluations at six-month intervals. The ability of the patient to walk with or without an assistance device was recorded in the last follow-up. The evaluation of function was graded according to the MSTS score (9). The oncological outcomes were assessed clinically using clinical examinations and imaging studies.

### Statistical analysis

A statistical analysis was implemented using SPSS 16.0 software (SPSS, Chicago, IL, USA). The comparisons of the variables comprised of continuous data were performed using two-sample t-tests. The comparisons of the discrete variables were performed using χ^2^ tests. P<0.05 was considered to indicate a statistically significant difference.

## Results

All tumor masses were removed completely without intraoperative shock or patient fatalities. The mean tumor size was 17.5 cm (range, 11.5–28 cm) at the greatest diameter. The average duration of the embolization procedure was 35 min (range, 20–70 min). The mean number of embolized vessels was 13.5 branches (range, 7–26 branches). The duration of the surgery was 2.5–8 hours, with an average time of 4.2 h. The average level of intraoperative blood loss was 1,293 ml (range, 400–4,500 ml). The average level of blood loss in the patients with malignant schwannomas (1,850 ml) was greater than in patients with benign neurogenic tumors (1,041.7 ml; P<0.05).

**Figure 1.**
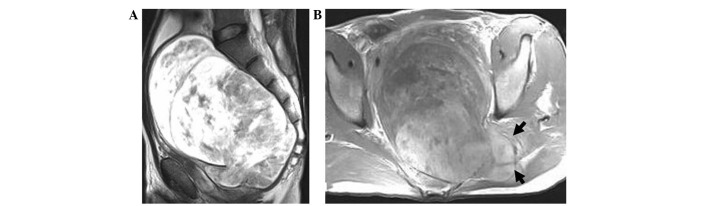
A 45-year-old male with a giant sacral malignant peripheral nerve sheath tumor (MPNST). MRI showing a large mass occupying the entire pelvic cavity. (A) The upper limit of the tumor reached the lower margin of L5 (T1-weighted MRI, sagittal view). (B) The lesion invaded the left gluteus muscle (black arrow; T2-weighted MRI, axial view).

**Figure 2.**
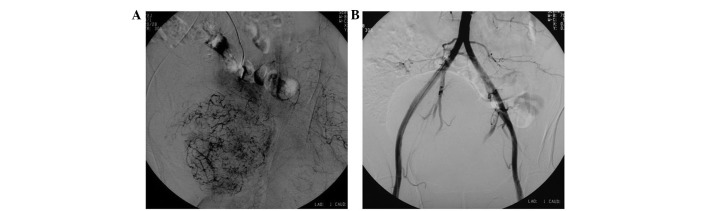
(A) The catheter was inserted into the internal iliac artery to perform an angiogram. (B) All tumor-feeding arteries were then completely embolized.

**Figure 3.**
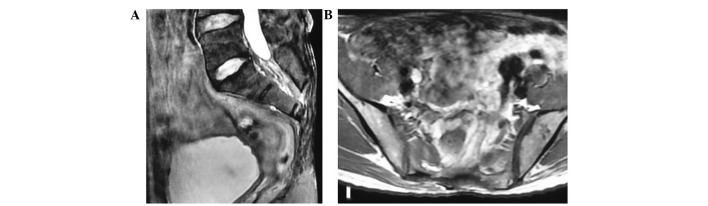
The tumor was marginally resected while preserving the bilateral S2 nerve roots. (A) MRI at the four-year follow-up showing no tumor recurrence (T1-weighted MRI, sagittal view). (B) Transverse view of MRI showing no recurrence in the surrounding muscle tissue (T2-weighted MRI, axial view).

The sacral nerve roots were preserved as much as possible in the patients with benign neurogenic tumors in order to maintain a greater neurological function. A marginal resection was performed in seven patients (58.3%) and the remaining five (41.7%) underwent intralesional resections due to extensive involvement of the sacral nerve roots. During surgery, the bilateral S1–S2 nerve roots, or at least the unilateral S3 nerve roots, were preserved in 11 patients. For the patients with MPNSTs, the affected sacral nerves were sacrificed in order to achieve adequate margins. As a result, one patient (25%) underwent a wide resection and three (75%) underwent marginal resections. The bilateral S1–S2 nerve roots, or at least the unilateral S3 nerve roots, were preserved in two patients; the unilateral S1–S2 nerve roots were preserved in one patient and the bilateral S1 nerve roots were preserved in the remaining patient. One of the patients with an MPNST had the largest tumor volume of ∼13×16×28 cm^3^ (Fig. 1). All the tumor-supplying vessels were embolized pre-operatively (Fig. 2) and the tumor was marginally resected while preserving the bilateral S2 nerve roots. The patient was disease-free at the four-year follow-up (Fig. 3). A pelvic reconstruction was performed in three of the 16 patients. No patients were treated pre- or post-operatively with adjuvant radiation therapy or chemotherapy. The mean hospital stay was 27 days (range, 15–48 days). Wound complications occurred in four patients (25%); three experienced cutaneous necrosis and one suffered a superficial wound infection. The surgical wounds of two of the patients healed with local wound care and debridement, and the wounds of the remaining two patients healed with flap closure and debridement. No patients experienced a clinical pulmonary embolism.

Overall, the average MSTS score of the 16 patients was 82% at the final follow-up. The score was 92.8% (range, 68–100%) in the benign cases, and 60.3% (range, 38.2–87.6%) for the patients with malignant disease. This difference was statistically significant (P<0.05). The MSTS scores of the patients with preservation of the bilateral S1/S2 nerve roots, or at least the unilateral S3 nerve roots (89%) were better than the scores of the patients in whom the bilateral S3 nerve was sacrificed (46%; P<0.05). The patients >40 years of age had a poor functional score compared with the patients <40 years, but the difference was not statistically significant (P>0.05).

Of the 12 patients with benign neurogenic tumors, 10 (83.3%) had normal bladder function and 11 (91.6%) had normal bowel control. For the patients with MPNSTs, two (50%) experienced impaired bladder control and two (50%) had impaired bowel control. Finally, two patients (12.5%) were managed by colostomy and three patients (18.8%) received intermittent urethral catheterization. All patients were able to ambulate independently and showed no signs of significant lower extremity weakness.

Follow-up information was obtained for all patients. The mean follow-up time was 59 months (range, 24–110 months). The oncological outcomes are presented in Table II. Recurrence or mortality as a result of the disease did not occur in the patients with benign tumors. The survival rate of the patients who presented with malignant lesions was 75% (3/4 patients), since 1 patient (25%) had multiple local recurrences and succumbed to the disease. However, no pulmonary or other metastases were identified in this patient.

## Discussion

Giant sacral neurogenic tumors are uncommon and their true incidence is unknown. However, they are estimated to represent 10% of the total presacral tumors (1) The majority of giant sacral neurogenic tumors are benign, with malignant schwannomas being rare. The literature on the surgical management of these tumors is limited to case reports and small studies (2–4). The growth of these tumors is frequently indolent and the lack of specific symptoms and signs make an early diagnosis difficult. The tumors often reach considerable sizes and may involve surrounding structures, making their management a surgical challenge.

The treatment of giant sacral neurogenic tumors remains controversial, since the main objective of surgical treatment is to achieve a complete surgical resection (4,6,10). However, the extent of the surgical resection is frequently limited by extensive intraoperative blood loss, particularly in giant MPNSTs (7,11). Methods to control the blood loss include occluding the abdominal aorta (6), ligation of the internal vein and artery (12), autologous blood transfusion (13) and manipulative hypotensive anesthesia. Nonetheless, studies have documented the mean level of blood loss during surgery as 1,600–4,300 ml (3,4,6). In the present study, a gelatin sponge was used as an embolic agent. The intratumor blood supply vessels and the main stem of the extratumor artery, including the bilateral internal iliac and median sacral arteries, were embolized completely. The level of blood transfusion averaged 1,293 ml (range, 0–4,000 ml) during the surgery. The level of blood loss was significantly decreased compared with previous data (3,4,6).

Several surgical approaches, including anterior, posterior and combined approaches, are used to resect sacral neurogenic tumors (4,6,14). The choice of approach is dictated by the location and size of the mass and its association with adjacent structures. For malignant pelvic tumors, Yokoyama *et al* (15) suggested that a patient's chances of definitive surgery may be enhanced by a multidisciplinary approach. Generally, the posterior approach is recommended for tumors that are situated at the third sacral (S3) segment or below. A combined anterior and posterior approach is advised for lesions that extend to the S1 or S2 segments. The decision to use only a posterior approach on patients in the present study is controversial. In our experience, the tumor rarely invades the rectal wall due to the presence of the presacral fascia, which resists tumor transgression. It is relatively easy to separate the anterior tissues using blunt dissection through the retroperitoneal interstitial spaces. In the present study, all tumor masses were removed completely without intraoperative shock or fatalities. Wide or marginal resection was performed on the four patients with MPNSTs. A posterior approach may be considered satisfactory for the resection of sacral neurogenic tumors, including those above S3, when combined with embolization. Moreover, pre-operative arterial embolization may have the potential to assist surgeons in completing resections using only a posterior approach.

Previous studies on sacral neurogenic tumors have focused on the oncological results without addressing the functional outcomes (4,6). Alderete *et al* (7) studied 38 patients with pelvic neurogenic tumors. Of these patients, twelve presented with malignant tumors. The patients with benign tumors had a mean MSTS score of 94%, while the survivors of malignant disease had a mean score of 57%. The majority of patients with benign tumors in the present study had higher MSTS scores and a better functional outcome when compared with the patients with malignant tumors, which is consistent with the results from the previous studies. Moreover, the results from the present study demonstrated that the preservation of the bilateral S1–S2, or at least the unilateral S3, nerve roots is significant in maintaining normal function.

The oncological results of sacral neurogenic tumors, particularly MPNSTs (7), are not as promising as those observed for the limbs. Due to the aggressive nature of MPNSTs, the overall survival rate is worse than for benign tumors. In the present study, the recurrence rate of MPNSTs was 25% (1/4 patients), which is comparable to the recurrence rates (40–71.4%) in the literature (6,7). No tumor recurrence or patient fatalities occurred in any of the benign cases. The prognosis for a sacral neurogenic tumor has been reported to be associated with tumor size, surgical margins or having had a prior surgical procedure (11). Sacrectomy has been suggested for treating giant sacral schwannomas (3,5). However, achieving a wide margin may sacrifice the normal adjacent neurovascular structures and associated function. Conversely, Pongsthorn *et al* (4) observed a good outcome with the use of a piecemeal subtotal excision in six giant sacral schwannoma cases. For benign sacral neurogenic tumors, local recurrences and malignant transformations are rare. Therefore, marginal or intralesional resections may be considered in order to preserve the sacral nerve roots and function. In the present study, adequate surgical treatment was performed for the MPNSTs. At the final follow-up, three patients were alive with no evidence of disease (NED) and one patient succumbed to repeated local recurrences.

The limitations of the present study include its retrospective nature, the relatively small numbers of patients and the use of a heterogenous group of patients with malignant and benign sacral neurogenic tumors. Despite these limitations, the present study may assist surgeons in recognizing the behavior of sacral neurogenic tumors. The results demonstrate that a surgical resection utilizing a posterior approach with pre-operative embolization is a viable treatment option for giant sacral neurogenic tumors.

## Figures and Tables

**Table I.** Chief complaint at presentation.

Complaint	Frequency, n (%)
Pain	7 (43.75)
Urinary disturbance or rectal dysfunction	4 (25.00)
Palpable painless mass	3 (18.75)
Neurological deficit	1 (6.25)
None (health examination)	1 (6.25)

**Table II.** Surgical type and outcome of 16 cases of sacral neurogenic tumors.

Tumor type	Type of surgery			Local recurrence	Metastasis	NED	AWD	Mortality
	Wide	Marginal	Intralesional					
Benign, n	0	7	5	0	0	10	2	0
Malignant, n	1	3	0	1	0	3	0	1

NED, no evidence of disease; AWD, alive with disease.
